# Differential diagnosis between pemphigoid and erosive lichen planus

**DOI:** 10.1590/1678-7757-2021-0657.res

**Published:** 2022-04-20

**Authors:** Ebru Saglam, Zeliha Betul Ozsagir, Tugba Unver, Suzan Bayer Alinca, Ali Toprak, Mustafa Tunali

**Affiliations:** 1 Health Sciences University Faculty of Dentistry Department of Periodontology Istanbul Turkey Health Sciences University, Faculty of Dentistry, Department of Periodontology Istanbul, Turkey.; 2 Bezmialem Vakif University Faculty of Dentistry Department of Maxillofacial Radiology Istanbul Turkey Bezmialem Vakif University, Faculty of Dentistry, Department of Maxillofacial Radiology, Istanbul, Turkey.; 3 Kecioren Osmanli Public Oral Health Center Ankara Turkey Kecioren Osmanli Public Oral Health Center, Oral and Maxillofacial Surgery, Ankara, Turkey.; 4 Bezmialem Vakif University Faculty of Medicine Department of Biostatistics and Medical Informatics Istanbul Turkey Bezmialem Vakif University, Faculty of Medicine, Department of Biostatistics and Medical Informatics, Istanbul, Turkey.; 5 Canakkal Onsekiz Mart University Faculty of Dentistry Department of Periodontology Canakkale Turkey Canakkal Onsekiz Mart University, Faculty of Dentistry, Department of Periodontology, Canakkale, Turkey.

We would like to thank you for the letter titled “Differential diagnosis between pemphigoid and erosive lichen planus” about our recent paper and the opportunity to respond.

We carefully read the letter and think that the raised argument is very important. Pemphigoid is a disease in which the epithelium separates from the connective tissue due to autoantibodies attacking basal membrane components. When only the mucous membranes are affected, the term benign mucous membrane pemphigoid (BMMP) is often used. The main diagnostic feature of BMMP is separation of the epithelium from the connective tissue at the basal membrane region. Pemphigoid should be diagnosed based on clinical evidence with immunohistochemical investigation.^1^ In a study of patients with the non-scarring phenotype of oral pemphigoid, circulating antibodies against the BP180 molecule were detected in 75% of cases.^2^ In our study, pemphigoid was considered in the differential diagnosis of lichen planus. In the case whose pictures were shared, the Nikolsky sign was negative, an air bubble test was performed, and no bullae formation was observed. Biopsy material taken from the lesion area and healthy mucosa were sent to histopathological testing. With the direct immunofluorescence method, linear weak IgG (+) deposition was observed along the dermo-epidermal junction. Immune complex deposition with IgM (-), IgA (-), C3 (-) and fibrinogen (-) was not detected, and the diagnosis of benign mucosal pemphigoid was excluded.

Oral lichen planus is microscopically characterized by three main features: hyperkeratosis or parakeratosis, hydropic degeneration of the basal layer, and dense, band-like infiltration of primarily T lymphocytes in the lamina propria. Hydropic degeneration of the basal layer of the epithelium may expand enough to cause the epithelium to become thinner and atrophic or separate from the underlying connective tissue, thus forming a subepithelial vesicle or ulcer.^3^ In the histopathology of the displayed case, the following were observed in the oral mucosa: Hyperkeratosis and parakeratosis proliferation in the stratified squamous epithelium; lymphocytes, apoptotic cells, hydropic degeneration in the basal layer; and in the subepithelial area, band-like infiltration of lymphocytes, histiocytes, and rare eosinophils, fibrosis, and abundant pigment-laden macrophages. No specific features were observed for PAS-Alcian blue and Giemsa.

**Figure 1:**
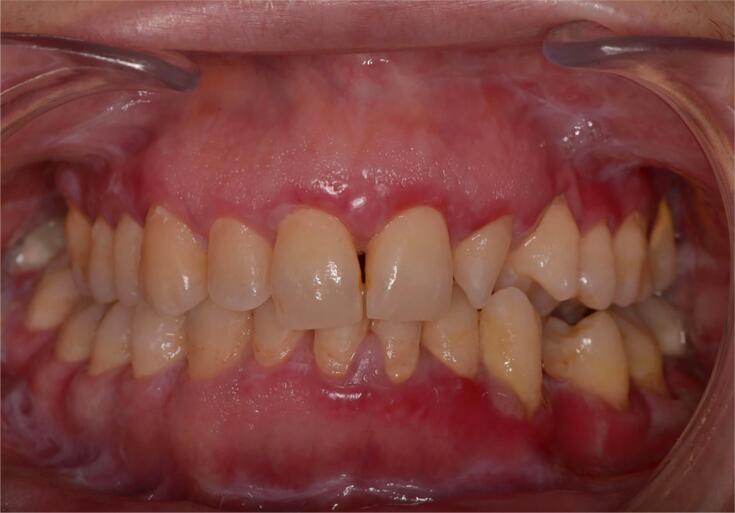
White striations seen on patient’s intraoral examination

If oral lichen planus is limited to gingival tissues (erosive oral lichen planus), the identification of thin, white spreading lines surrounding the erosive areas supports the clinical diagnosis of oral lichen planus. If white striae are absent, the differential diagnosis should primarily include mucous membrane pemphigoid and pemphigus vulgaris.^3^ White striae were also observed in the patient’s intraoral examination (Figure 1). White striae are absent in pemphigoid, as the authors stated in the letter. Since there was a distinct correlation between histopathological and clinical findings, the case was considered to be erosive lichen planus.
